# Single and fractionated ionizing radiation induce alterations in endothelial connexin expression and channel function

**DOI:** 10.1038/s41598-019-39317-9

**Published:** 2019-06-20

**Authors:** Raghda Ramadan, Els Vromans, Dornatien Chuo Anang, Elke Decrock, Mohamed Mysara, Pieter Monsieurs, Sarah Baatout, Luc Leybaert, An Aerts

**Affiliations:** 1 Radiobiology Unit, Belgian Nuclear Research Centre (SCK·CEN), Mol, Belgium; 2 Department of Basic and Applied Medical Sciences, Physiology group, Ghent University, Ghent, Belgium; 3 Centre for Environmental Health Sciences, Hasselt University, Hasselt, Belgium; 4 Biomedical Research Institute and transnational university of Limburg, Hasselt University, Hasselt, Belgium; 5 Microbiology Unit, Belgian Nuclear Research Centre (SCK·CEN), Mol, Belgium; 6 Department of Molecular Biotechnology, Ghent University, Ghent, Belgium

**Keywords:** Molecular biology, Atherosclerosis

## Abstract

Radiotherapy is an effective treatment for most tumor types. However, emerging evidence indicates an increased risk for atherosclerosis after ionizing radiation exposure, initiated by endothelial cell dysfunction. Interestingly, endothelial cells express connexin (Cx) proteins that are reported to exert proatherogenic as well as atheroprotective effects. Furthermore, Cxs form channels, gap junctions and hemichannels, that are involved in bystander signaling that leads to indirect radiation effects in non-exposed cells. We here aimed to investigate the consequences of endothelial cell irradiation on Cx expression and channel function. Telomerase immortalized human Coronary Artery/Microvascular Endothelial cells were exposed to single and fractionated X-rays. Several biological endpoints were investigated at different time points after exposure: Cx gene and protein expression, gap junctional dye coupling and hemichannel function. We demonstrate that single and fractionated irradiation induce upregulation of proatherogenic Cx43 and downregulation of atheroprotective Cx40 gene and protein levels in a dose-dependent manner. Single and fractionated irradiation furthermore increased gap junctional communication and induced hemichannel opening. Our findings indicate alterations in Cx expression that are typically observed in endothelial cells covering atherosclerotic plaques. The observed radiation-induced increase in Cx channel function may promote bystander signaling thereby exacerbating endothelial cell damage and atherogenesis.

## Introduction

Although radiotherapy is a common effective treatment for most tumour types, it also results in an increased risk for developing radiation-related side effects such as cardiovascular diseases (CVD)^1–7^. Radiation exposure to the cardiovascular system occurs during radiotherapy for cancer treatment in the thoracic region, such as breast cancer, head-and-neck cancer and Hodgkin’s lymphoma^8^. Indeed, epidemiological data clearly indicate an excess risk of late occurring CVD, especially atherosclerosis, after ionizing radiation (IR) exposure^2,6,9–12^. However, the underlying cellular and molecular mechanisms are not fully understood, possibly resulting in improper radiation protection.

Atherosclerosis is a progressive inflammatory disease of the arterial wall that is initiated with damage to the vascular endothelial cells. IR exposure induces endothelial effects such as DNA damage, oxidative stress, apoptosis, inflammation, senescence and cellular Ca^2+^ overload, which induce endothelial cell dysfunction and link the radiation exposure to the pathogenesis of atherosclerosis^4,8–10,13–18^.

Cellular and molecular changes induced by IR occur not only in directly irradiated, but also in adjacent non-irradiated cells, a process known as ‘the bystander effect’^19,20^. Transmembrane connexin proteins (Cxs) are suggested to play an important role in this process by forming intercellular gap junctional and hemichannel paracrine communication pathways^20^. There are three different Cx isotypes expressed in endothelial cells of the major arteries, namely Cx37, Cx40 and Cx43. Growing evidence indicates that Cxs play a role in the pathology of atherosclerosis^21–24^. Although healthy vascular endothelial cells mainly express Cx37 and Cx40, both Cxs are lost in the endothelium covering advanced atherosclerotic plaques^22,25^. In contrast, Cx43 typically has a low expression in the healthy endothelium, reported to increase the formation of atherosclerotic lesions *in vivo*^26,27^, and becomes clearly detectable at specific regions of advanced atherosclerotic plaques^21–23^. The mechanisms responsible for Cx modifications in atherosclerosis are not fully understood. However, it has been demonstrated that Cx37 is a regulator of endothelial NO synthase (eNOS) expression and function^28^. Decreased Cx37 expression induces downregulation of eNOS and decreased NO vasodilatory signaling^28,29^, impairing the regulation of vascular tone^30^. Moreover, Cx37 may protect against atherosclerosis development by regulating monocyte adhesion (46). Therefore, Cx37 may act in an atheroprotective manner. Cx40 may act in a similar manner: endothelial-specific deletion of Cx40 results in proatherogenic by increasing CD73-dependent leukocyte adhesion to the endothelium^31^ and decreased endothelial NO signaling^29^. In addition Cx40-mediated gap junctional communication may contribute to a quiescent non-activated endothelium by generating anti-inflammatory signals between endothelial cells^31^. Moreover, endothelial-specific deletion of Cx40 induced neutrophil infiltration, increased cell death and increased infarct size in mice following ischemia-reperfusion injury^32^. In contrast to the atheroprotective effects of Cx37 and Cx40, Cx43 is endowed with proatherogenic properties^26,33^. Downregulation of Cx43 expression inhibits monocyte-endothelial adhesion by decreasing the expression levels of cell adhesion proteins including VCAM-1, while Cx43 upregulation enhanced these cell adhesion proteins^34^. Interestingly, irradiation of human fibroblasts with 10 mGy of α-particles induces an upregulation of Cx43 expression^35^, and low dose 137Cs source irradiation increases Cx43 expression in human fibroblasts^36^. Moreover, the level of Cx43 was significantly elevated in mouse endothelial cell line bEnd3 exposed to 5 Gy of X-rays^37^.

Although Cxs have been reported to be sensitive to IR and to be involved in atherosclerosis pathogenesis, their role in radiation-induced endothelial cell responses were never investigated before. In particular, coronary artery and microvascular endothelial cells may act in concert to produce cardiovascular complications via combined macrovascular and microvascular post irradiation injury^38^. Here, we aimed at investigating alterations in endothelial Cx expression and channel function in response to IR. We report, for the first time, that single and fractionated X-ray irradiation modulate coronary artery and microvascular endothelial Cx gene expression, protein levels and channel function that may possibly act in a proatherogenic manner.

## Results

### Single and fractionated irradiation induce changes in the gene expression of atheroprotective Cx37 and Cx40 and proatherogenic Cx43

#### Single irradiation

TICAE and TIME cells were exposed to different single doses of X-rays (0.1, 0.5 and 5 Gy) and assessed for changes in Cx gene expression at different time points (6 h, 24 h, 48 h, 72 h, 7 d and 14 d p.i.).

**Figure 1:**
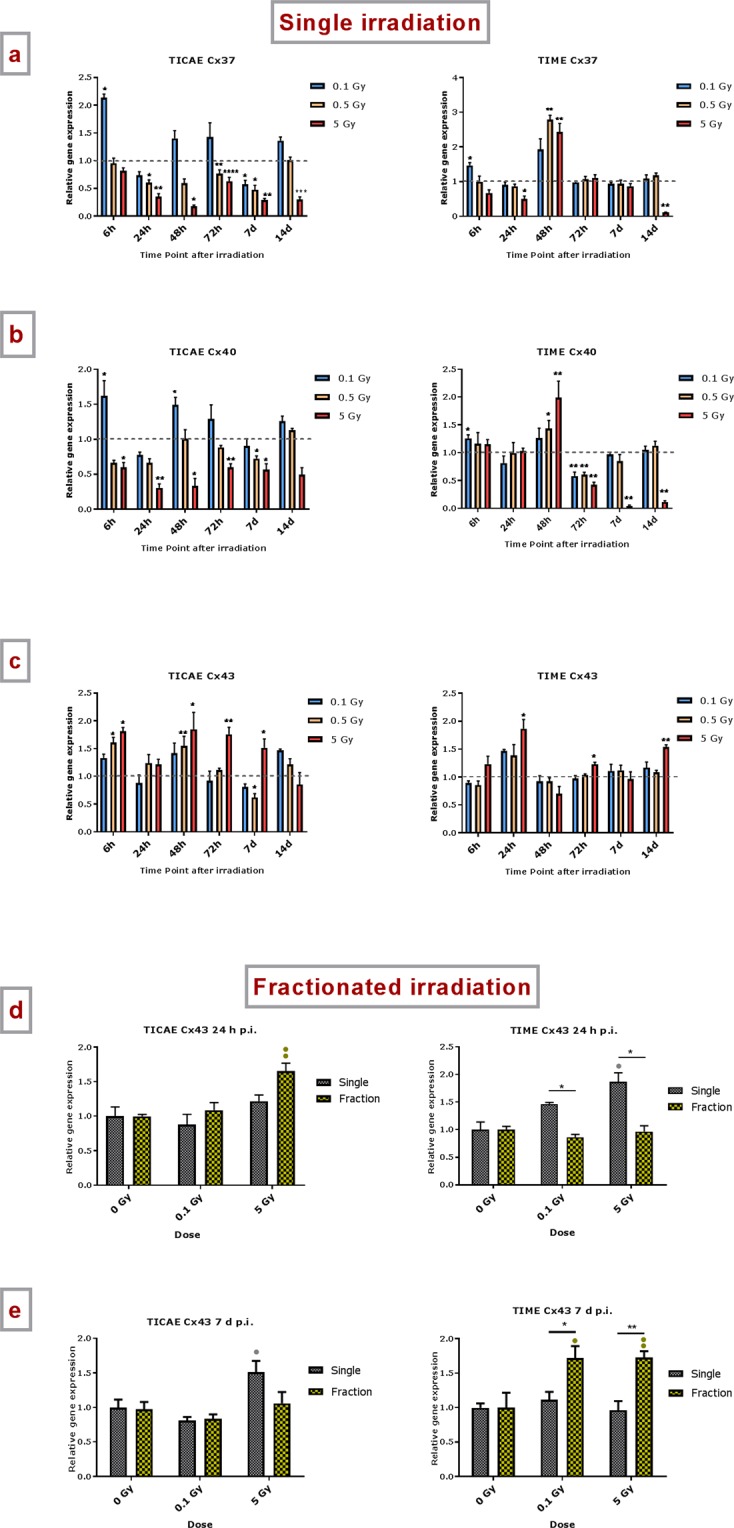
The effect of single and fractionated irradiation on Cx37, Cx40 and Cx43 gene expression. Gene expression of Cx37, Cx40 and Cx43 at 6 h, 24 h, 48 h, 72 h, 7 d or 14 d after a single X-ray exposure (0.1, 0.5 and 5 Gy) in TICAE (left side) and TIME cells (right side) (a,b,c). Gene expression of Cx37, Cx40 and Cx43 at 24 h (d) and 7 d (e) after single or fractionated irradiation in TICAE (left side) and TIME cells (right side). Fractionated irradiation involved three consecutive X-rays doses (0.033 and 1.67 Gy/fraction/day), leading to cumulative doses of 0.1 and 5 Gy. Data were analyzed with a nonparametric Mann-Whitney T-test. Values represent average ± SEM of 5 biological replicates, except for 6 h p.i. where 4 biological replicates were used. (**a**–**c**) *Indicates for a given time point the statistical difference of gene expression after a dose of single irradiation compared to the respective normalized 0 Gy controls at the same time point. (**d**–**e**) ^•^Indicates for a given time point the statistical difference of gene expression after a dose of fractionated irradiation compared to the respective normalized 0 Gy controls at the same time point. (**d**–**e**) *Indicates the statistical difference between fold changes of gene expression after a given radiation dose and a given time of single and fractionated irradiation compared to the respective normalized 0 Gy controls at the same time point. ^*/•^p < 0.05; ^**/••^p < 0.01; ^***/•••^p < 0.0001. Cx, connexin; TICAE, Telomerase Immortalized human Coronary Artery Endothelial cells; TIME, Telomerase Immortalized human Microvascular Endothelial cells; p.i, post irradiation; h, hours; d, days; SEM, standard error of mean.

In TICAE cells, a radiation-induced downregulation in Cx37 gene expression was observed starting from 24 h p.i., which persisted up to 14 d p.i. and were mainly significant for the 0.5 Gy and 5 Gy doses. For 0.1 Gy exposures, Cx37 gene expression was significantly upregulated at an early time point (6 h p.i.), while downregulated at a late time point (7 d p.i.) (Fig. 1a). In TIME cells, 5 Gy irradiation induced downregulation of Cx37 gene expression at 24 h and 14 d p.i., while upregulation was observed at 48 h. Irradiation at 0.1 and 0.5 Gy gave upregulation at 6 h and 48 h p.i. respectively (Fig. 1a).

Gene expression of Cx40 was downregulated in TICAE cells starting from 6 h and persistent up to 7 d p.i., mainly significant at 5 Gy (Fig. 1b). For 0.1 Gy exposures, Cx40 gene expression showed a significant upregulation at 6 h and 48 h p.i.. In TIME cells, Cx40 gene expression showed an upregulation at early time points, significant at 6 h for 0.1 Gy, and at 48 h for 0.5 Gy and 5 Gy. At 72 h p.i., a dose-dependent downregulation was observed, which persisted up to 7 and 14 d for the 5 Gy dose (Fig. 1b).

Different from Cx37 and Cx40, irradiation mainly resulted in an upregulation of Cx43 gene expression for the 0.5 and 5 Gy doses. In TICAE cells, 5 Gy induced significant upregulation of gene expression at 6 h and 48 h p.i., which persisted up to 7 d p.i. For the 0.5 Gy dose, upregulation normalized more rapidly (Fig. 1c). In TIME cells, 5 Gy caused significant upregulation at 24 h, 72 h and 14 d p.i., while lower doses did not induce significant alterations.

#### Fractionated irradiation

An early and late time point (24 h and 7 d p.i.) were selected to evaluate the effect of fractionated irradiation on Cx gene expression, and to compare it to single exposure effect (by comparing the relative gene expression (fold change) between single and fractionated exposure to their respective normalized controls).

For Cx37, fractionated irradiation of TICAE cells resulted in a downregulation of gene expression, which was significant for 5 Gy at both time points and for 0.1 Gy only at the 7 d time point, like single dose irradiation (Supplementary Fig. S1a,b). In line with the similar effect of both dose regimens, no significant differences were found between single and fractionated irradiation. In TIME cells, Cx37 gene expression was found to be significantly downregulated for the 5 Gy fractionated regimen at the 24 h time point only, as in single irradiation, but surprisingly the 7 d time point showed strongly significant upregulation. As expected from this strong upregulation, the response to fractionated 0.1 and 5 Gy irradiation was significantly higher as compared to single irradiation (Supplementary Fig. S1b).

For Cx40, fractionated irradiation of TICAE cells resulted in significant downregulation of gene expression for the 5 Gy dose at 24 h, like single irradiation, but not at 7 d, unlike single irradiation, resulting in significant differences for the two radiation regimens at 5 Gy for the 7 d time point (Supplementary Fig. S1c,d). In TIME cells, Cx40 gene expression was significantly downregulated at 5 Gy at the 24 h time point, and at 0.1 Gy and 5 Gy at the 7 d time point. In accordance, there is a significant difference between single and fractionated irradiation on Cx40 gene expression at 5 Gy at 24 h p.i. and at 0.1 Gy at 7 d p.i. (Supplementary Fig. S1c,d).

For Cx43, fractionated irradiation of TICAE cells induced significant upregulation of gene expression at the 24 h time point and things recovered at the 7 d time point, but there were no significant differences for the two radiation regimens (Fig. 1d,e). In TIME cells, fractionated irradiation did not induce changes in Cx43 gene expression after 24 h, unlike observations with single irradiation where 5 Gy caused upregulation. By contrast, at the 7 d time point, fractionated irradiation upregulated Cx43 gene expression (significant for both 0.1 and 5 Gy doses) while a single dose had no effect. As expected from the diverse effects of single *vs* fractionated irradiation, comparisons between the two dose delivery schemes showed several significant differences for the 24 h and 7 d time points as well as for the 0.1 and 5 Gy doses (Fig. 1d,e). Taken together with the observations for Cx37 and Cx40, these experiments clearly demonstrate distinct effects of fractionated irradiation *vs* single irradiation in TIME cells. For Cx40 and Cx43, the direction of the difference seems to depend on the time point after irradiation.

### Single and fractionated irradiation decrease atheroprotective Cx40 protein expression and increase proatherogenic Cx43 protein expression

#### Single irradiation

TICAE and TIME cells were exposed to different single doses of X-rays (0.1, 0.5 and 5 Gy) and assessed for changes in Cx protein levels at different time points (6 h, 24 h, 48 h, 72 h, 7 d and 14 d p.i.). Expression of Cx37 protein could not be detected with the 10–30 µg protein concentration used, indicating low endogenous levels.

**Figure 2:**
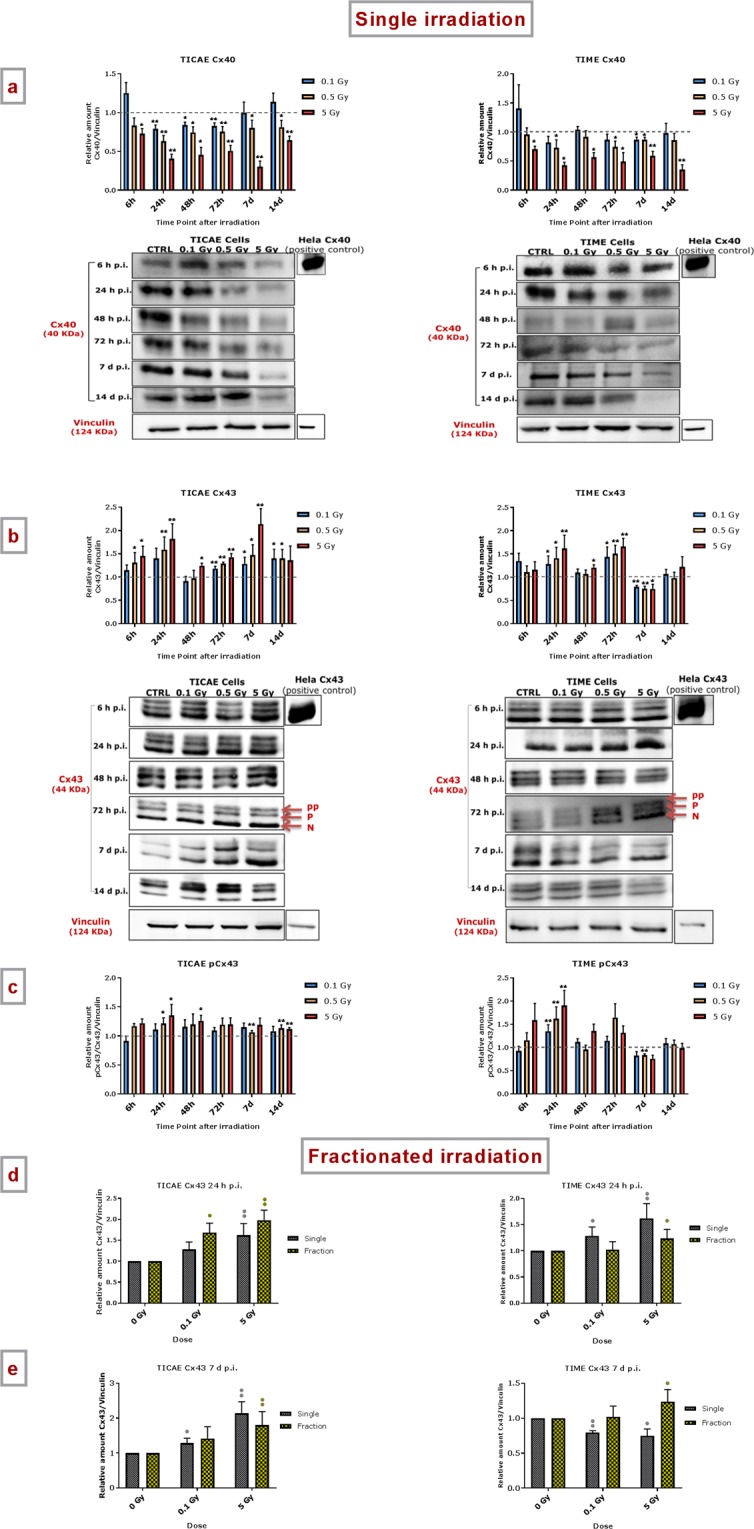
The effect of single and fractionated irradiation on Cx40, Cx43 and pCx43 protein levels. Cx40, Cx43 and pCx43 protein levels were assessed 6 h, 24 h, 48 h, 72 h, 7 d and 14 d after a single X-ray exposure (0.1, 0.5 and 5 Gy) in TICAE (left side) and TIME cells (right side) relative to 0 Gy controls (a,b,c). Cropped blots are represented below each graph (a,b) and full-length blots are reported in Supplementary Fig. S4. All gels were run following the same experimental conditions (see methods for details). Hela cells overexpressing Cx43 or Cx40 were used as a positive control for assessing protein levels of Cx43 or Cx40, respectively. Signals were normalized to the corresponding vinculin signal of the same membrane and quantified densitometrically using Bio1D analysis software. Single and fractionated irradiation 24 h (d) and 7 d (e) post irradiation on Cx43 protein level in TICAE (left side) and TIME cells (right side). Data were analyzed with a nonparametric Mann-Whitney T-test. Values represent average ± SEM of 4–6 biological replicates. (**a–c**) *Indicates the statistical differences compared to the respective 0 Gy controls at the same time point, (**d,e**) *Indicates the statistical differences between single and fractionated irradiation for the same radiation dose. ^•^Indicates the statistical differences for either single or fractionated irradiation compared to their respective 0 Gy controls ^*/•^p < 0.05; ^**/••^p < 0.01; ^***/•••^p < 0.0001.

A radiation-induced acute and persistent decrease in Cx40 protein level was observed in a dose-dependent manner in both TICAE and TIME cells (Fig. 2a). In TICAE cells, only the 5 Gy dose demonstrated significantly decreased Cx40 expression at the early time point of 6 h. However, in the period between 24 h and 72 h all doses showed significantly decreased Cx40 protein levels; this decrease was continued up to 14 d p.i. for the 0.5 and 5 Gy doses. In TIME cells, the decrease in Cx40 protein level was significant at all time points for 5 Gy; for 0.5 Gy significant decrease was attained at 24 h, 72 h and 7 d p.i., while for 0.1 Gy significance was only attained at 7 d p.i. (Fig. 2a).

We found a radiation-induced acute and persistent increase in Cx43 protein level that was dose-dependent for both TICAE and TIME cells (Fig. 2b). For the 5 Gy dose, the increase was significant for all time points except for 14 d p.i.; for the 0.5 Gy dose, all time points except 48 h were significant; for 0.1 Gy, a significant increase was apparent from 72 h on, which remained high up to 14 d p.i.. In TIME cells, significant increases in Cx43 protein levels were mainly concentrated in the 24 h to 72 h time window, and at 7 d p.i., a slight, but significant decrease in Cx43 protein level was observed (Fig. 2b).

In addition, a radiation-induced increase in the phosphorylated (p) and hyperphosphorylated (pp) forms of Cx43 were observed in TICAE cells at 24 h, 48 h, 7 d and 14 d p.i. (mainly significant at 0.5 Gy and 5 Gy) (Fig. 2c). In TIME cells, a dose-dependent increase in the phosphorylated and hyperphosphorylated forms was observed mainly at 24 h p.i. (Fig. 2c).

#### Fractionated irradiation

An early and late time point (24 h and 7 d p.i.) were selected to evaluate the effect of fractionated irradiation on Cx protein expression, and to compare it to single exposure effect.

For Cx40, fractionated irradiation of TICAE cells induced a significant decrease in the protein level only for the 5 Gy dose at the 24 h and 7 d time points, but the effects were less pronounced compared to single irradiation at 7 d time points (Supplementary Fig. S2a,b). In TIME cells, fractionated irradiation induces a significant decrease at 5 Gy in Cx40 protein level at 24 h time point, like single irradiation, and then stabilizes at 7 d post fractionated irradiation, unlike single irradiation. In accordance, there is a significant difference between single and fractionated irradiation on Cx40 protein level at 5 Gy after 7 d of the exposure (Supplementary Fig. S2a,b).

For Cx43, fractionated irradiation of TICAE cells induced an increase in the protein level, which was most clear at the 24 h time point (significant for both 0.1 and 5 Gy doses) and less prominent at the 7 d time point (significant for 5 Gy only) (Fig. 2d,e). In TIME cells, fractionated irradiation induced an increase in Cx43 protein level at 5 Gy for both 24 h and 7 d time point, however, these effects were less pronounced than those observed in TICAE cells (Fig. 2d,e). Importantly, no significant differences were observed between single and fractionated irradiation in both TICAE and TIME cells.

### Single and fractionated irradiation exposure induce an increase in gap junction-mediated dye coupling

#### Single irradiation

**Figure 3:**
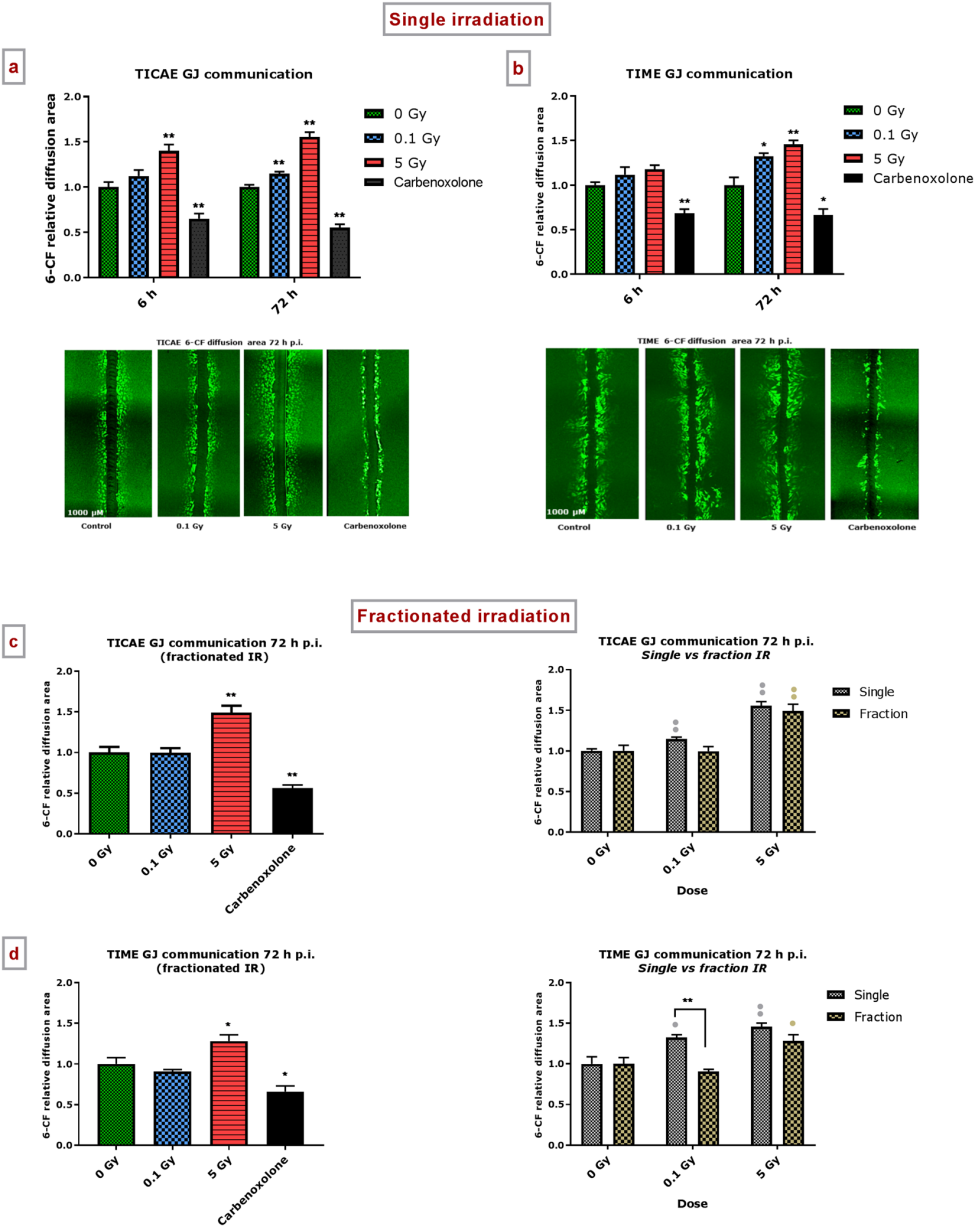
Single and fractionated radiation exposure induce an increase in gap junctional communication. The area of diffusion of the 6-CF dye, representing gap junctional communication was assessed after single irradiation exposure in (**a**) TICAE, (**b**) TIME cells, and after fractionated irradiation exposure in (**c**) TICAE cells and (**d**) TIME cells. Carbenoxolone was used as a control. Data were analyzed with a nonparametric Mann-Whitney T-test. Values represent average ± SEM of five to six biological replicates. *Indicates the statistical differences compared to the respective 0 Gy controls at the same time point, and the statistical differences between single and fractionated irradiation for the same radiation dose. ^•^Indicates the statistical differences for either single or fractionated irradiation compared to their respective 0 Gy controls. ^*/•^p < 0.05; ^**/••^p < 0.01. 6-CF, 6-carboxyfluorescein; GJ, gap junction.

After exposure of TICAE and TIME cells to a single dose of 0.1 or 5 Gy, SLDT assays were performed at 6 h and 72 h p.i.. At 6 h p.i., a significant increase in 6-CF diffusion area was observed for the 5 Gy dose in TICAE cells compared to control non-irradiated cells (Fig. 3a). At 72 h p.i., a significant increase in 6-CF diffusion area was observed for 0.1 and 5 Gy in both TICAE and TIME cells compared to control non-irradiated cells (Fig. 3a,b). Carbenoxolone inhibited the dye spread in all cases. These results reflect a radiation-induced increase in gap junction communication, even for low doses at 72 h p.i. in TICAE and TIME cells (summarized in Supplementary Table S1).

#### Fractionated irradiation

The 72 h p.i. time point was selected to evaluate the effect of fractionated irradiation on dye spread (fractionated irradiation as applied in the preceding experiments), and to compare it to single exposure effect. For TICAE and TIME cells, only the 5 Gy dose resulted in significantly increased dye spread (Fig. 3c,d, left panels). For TICAE cells, there were no differences between fractionated and single exposures (Fig. 3c, right panel). In TIME cells, fractionated irradiation with 0.1 Gy was significantly less potent in increasing dye spread compared to single irradiation (Fig. 3d, right panel).

### Radiation-induced alterations in hemichannel function

#### Assessment of hemichannel opening by measuring extracellular ATP release

We first assessed hemichannel opening by determining ATP release in response to 0.1 Gy and 5 Gy irradiation at 1 h, 6 h and 72 h p.i. and tested the effect of hemichannel blockade with TAT-Gap19 which blocks hemichannels composed of Cx43 while it does not inhibit gap junctions^33,39^, reviewed in^23^.

**Figure 4:**
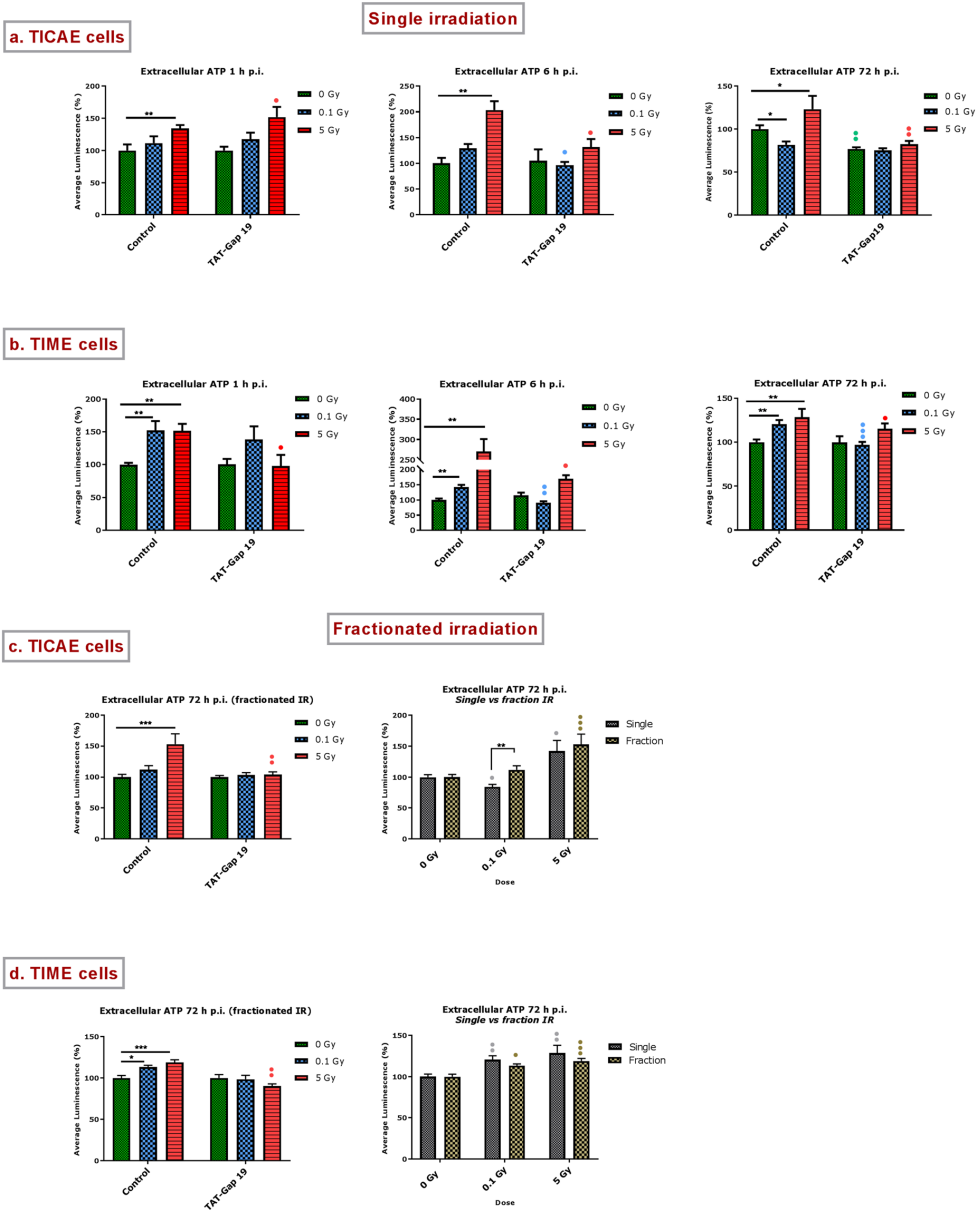
Radiation-induced increase in extracellular ATP and the effect of TAT-Gap19 after single and fractionated irradiation. ATP release was measured 1 h, 6 h and 72 h after single radiation exposure (0.1 and 5 Gy) in (**a**) TICAE cells and (**b**) TIME cells and 72 h after fractionated irradiation exposure in (**c**) TICAE cells and (**d**) TIME cells. TAT-Gap19 was used to block Cx43 hemichannels. Data were analyzed with a nonparametric Mann-Whitney T-test. Values represent average ± SEM of 6–8 biological replicates. *Indicates the statistical differences compared to the respective 0 Gy controls, and statistical difference between single and fractionated irradiation for the same radiation doses. ^•^Indicates the statistical difference compared to the respective control condition (not treated with TAT-Gap 19), (**c,d**) ^•^indicates the statistical differences for either single or fractionated irradiation compared to their respective 0 Gy controls; ^*/•^p < 0.05; ^**/••^p < 0.01; ^***/•••^p < 0.0001.

Single irradiation: In TIME cells, the effects were most strong and both 0.1 and 5 Gy doses triggered significant ATP release at all time points (1, 6 and 72 h) (Fig. 4b). In TICAE cells, only 5 Gy induced significant ATP release, and in all cases, except the 1 h p.i. case, TAT-Gap19 significantly reduced the ATP release (Fig. 4a). In most cases, TAT-Gap19 significantly inhibited the radiation-induced ATP release but no inhibition was observed for the 1 h p.i. time point for the 0.1 and 5 Gy doses in TICAE and the 0.1 Gy dose in TIME cells. This lack of TAT-Gap19 effect may indicate ATP release via pathways other than Cx43 hemichannels, which may predominate in the early 1 h period after irradiation. Supplementary Table S1 summarizes the responses to single irradiation that were inhibited by TAT-Gap19, thereby reflecting Cx43 hemichannel involvement.

Fractionated irradiation: The 72 h p.i. time point was selected to evaluate the effect of fractionated irradiation on ATP release, and to compare it to single exposure effect (Fig. 4c,d). As observed with single irradiation, TIME cells showed significant ATP release for both 0.1 and 5 Gy doses while responses in TICAE cells were only significant for 5 Gy. In both cells, TAT-Gap19 significantly inhibited the 5 Gy responses only, presumably because the responses at 0.1 Gy were either non-significant (TICAE) or small (TIME cells) (Fig. 4c,d, left panel). Interestingly, fractionated irradiation in TICAE cells induced significantly more ATP release at 0.1 Gy compared to single irradiation (Fig. 4c right panel).

#### Assessment of hemichannel opening via dye uptake assays

To further assess hemichannel opening, the previous experiments were complemented with dye uptake assays using propidium iodide (PI) and 10 kDa dextran fluorescein as reporter dyes. As an additional control, we also verified the effect of Cx43 hemichannel blockade with TAT-Gap19.

**Figure 5:**
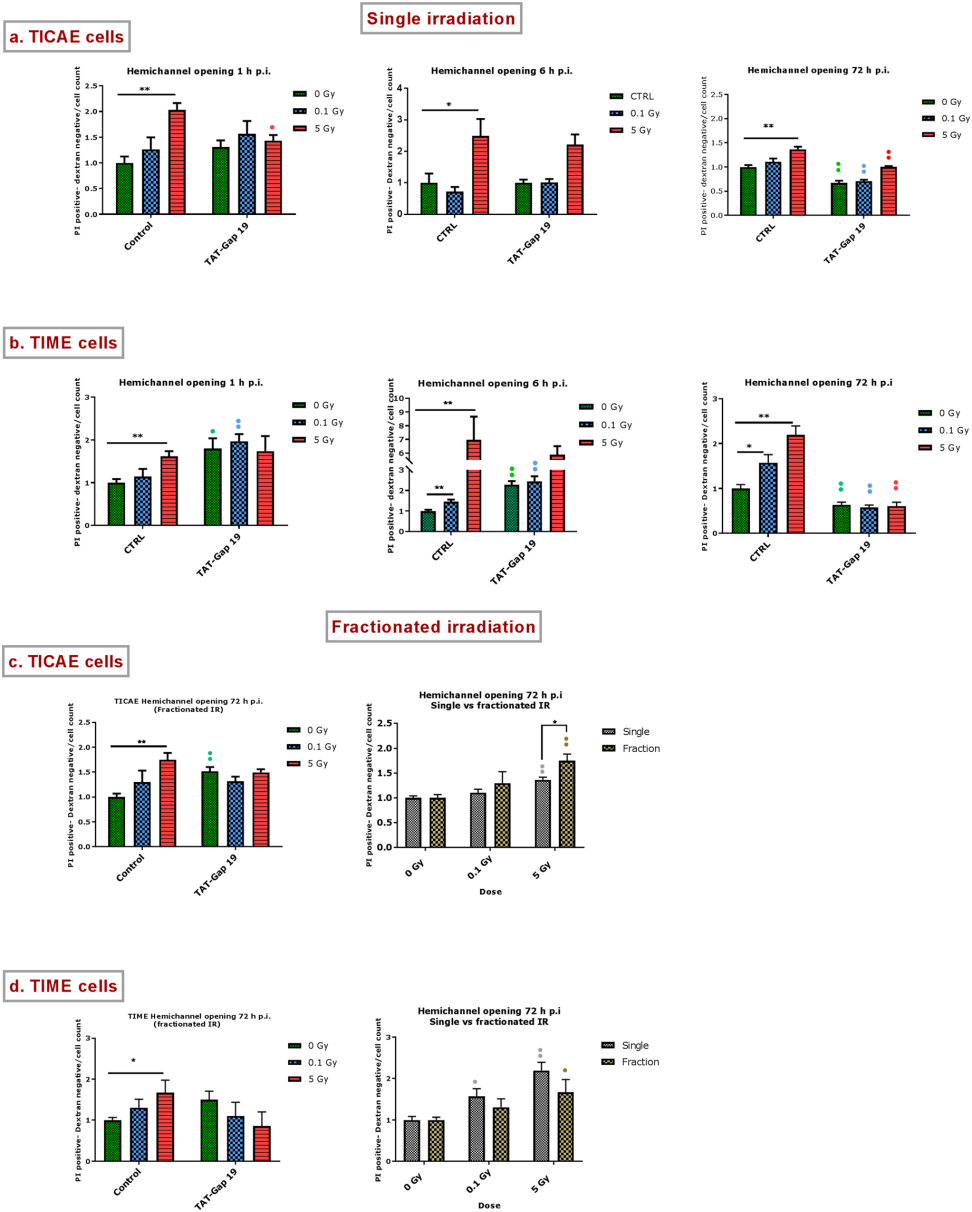
Radiation-induced PI dye uptake responses and the effect of TAT-Gap19 after single and fractionated radiation exposure. (**a**) Dye uptake 1 h, 6 h and 72 h p.i. in TICAE cells. (**b**) Dye uptake responses in TIME cells. (**c**) Dye uptake 72 h after fractionated irradiation in TICAE cells. (**d**) Dye uptake responses to fractionated irradiation in TIME cells. Data were analyzed with a nonparametric Mann-Whitney T-test. Values represent average ± SEM of 6 biological replicates. *Indicates the statistical differences compared to the respective 0 Gy controls, and statistical difference between single and fractionated irradiation for the same radiation doses. ^•^Indicates the statistical difference compared to the respective control condition (not treated with TAT-Gap 19), (**c,d**) ^•^Indicates the statistical differences for either single or fractionated irradiation compared to their respective 0 Gy controls. ^*/•^p < 0.05; ^**/••^p < 0.01.

Single irradiation: A radiation-induced increase in dye uptake was mainly significant for the 5 Gy dose at 1 h, 6 h and 72 h p.i. in both TICAE and TIME cells; in TIME cells, the lower 0.1 Gy dose also gave significant dye uptake (Fig. 5a,b). Collectively, these responses were very similar to those observed in the ATP release assays. TAT-Gap19 inhibition of these responses was most clear and significant at the 72 h p.i. time point for both cells. Significant inhibition by TAT-Gap19 was also seen at 1 h p.i. in TICAE cells exposed to 5 Gy. In line with the ATP release data, TAT-Gap19 was less efficient in inhibiting dye uptake in the early post irradiation period, here including both the 1 h and 6 h time points. This may indicate PI dye uptake via poorly selective channels other than Cx43 hemichannels and activated by irradiation. Supplementary Table S1 summarizes dye uptake responses that were inhibited by TAT-Gap19, thereby reflecting Cx43 hemichannel involvement.

Fractionated irradiation: The 72 h time point was selected to evaluate the effect of fractionated irradiation on PI dye uptake, and to compare it to single exposure effect. Fractionated irradiation induced an increase in dye uptake in TICAE and TIME cells exposed to 5 Gy but here TAT-Gap19 had no effect (Fig. 5c,d, left panels). Interestingly, TICAE cells exposed to fractionated 5 Gy irradiation induced significantly stronger dye uptake compared to a single irradiation scheme (Fig. 5c, right panel).

### Principal component analysis

**Figure 6:**
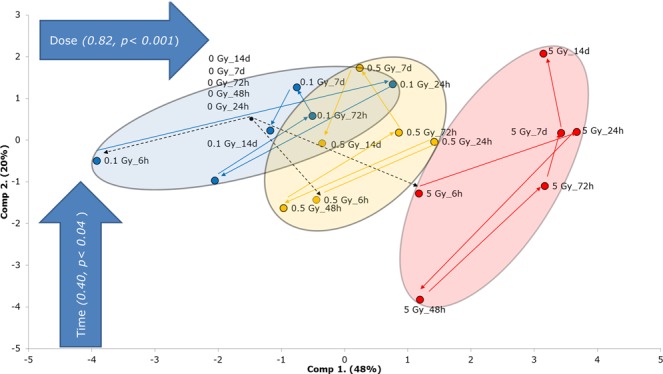
Principal component analysis (PCA) incorporating both Cx40 and Cx43 gene expression and protein level in TICAE and TIME cells from 6 h to 14 d time period. The colors correspond to the radiation doses, where 0.1, 0.5 and 5 Gy were colored blue, orange, red, respectively.

In order to have a concise view on the major effects observed, we performed principal component analysis (PCA) for Cx40 and Cx43 gene expression and protein level in both TICAE and TIME cells after single irradiation over the 6 h to 14 d p.i. time period (Fig. 6). This analysis indicated a dose-dependent separation between the radiation doses used (0.1, 0.5 and 5 Gy), which significantly shifted the PCA profiles along the positive side of the first component axis *(p* < *0.001)* reflecting dose-dependent alterations in Cx40 and Cx43. In particular, the dose significance started from the 0.5 Gy dose (*p* < *0.00017)* followed by the 5 Gy dose (*p* < *0.001)* all compared to the non-irradiated control. In addition, a time factor could explain the profile shifts of the irradiated cells along the positive side of the second component axis, with statistical significance (p < 0.04). In particular, the late time points group together with the non-irradiated control, i.e. the effect was neutralized and the neutralization effect apparently became more pronounced at 14 d for 0.1 Gy condition. On the other hand, such waxing and waning of the radiation effect was not observed for the 0.5 Gy condition and more pronounced for the 5 Gy condition where the profiles shifted away from the control up to the last measurement at 14 d post exposure (i.e. the effect was persistent) (Fig. 6).

## Discussion

Growing epidemiological data suggest that endothelial cell irradiation induces atherosclerosis^2,12^. However, the underlying mechanisms are not fully understood. Although connexins were reported to be sensitive to IR and to play a role in atherosclerosis development, their role in radiation-induced atherosclerosis has been poorly explored^22,35^. Here, we aimed to investigate changes in connexin expression and channel function in response to IR, as a putative mechanism leading to radiation-induced endothelial cell dysfunction, an early event in the atherosclerotic process. We report that single and fractionated X-ray irradiation of coronary artery and microvascular endothelial cells, at doses that can be received after serial diagnostic procedures (0.1 Gy) or as out-of-field exposure after radiotherapy (5 Gy), significantly alters endothelial connexin gene expression, protein levels, gap junctional dye coupling and hemichannel function. Below we discuss these findings in more detail.

We demonstrate changes in Cx37 gene level, consisting of an early (6 h) upregulation at 0.1 Gy followed by a dose-dependent downregulation in TICAE cells and fluctuating responses in TIME cells. These alterations indicate that Cx37 modulation in response to IR is cell line specific, possibly related to distinct endothelial properties at different sites of the vascular tree. For Cx40, gene expression was up at the early time point for the 0.1 Gy dose in the two cell types, as observed for Cx37. Higher doses mainly gave downregulation in TICAE cells and showed more variable responses in TIME cells. Interestingly, the early transient upregulation at 0.1 Gy for both Cx37 and Cx40 genes, observed in both cell types, may reflect possible early protective effects. Protective effects after low dose ionizing irradiation in endothelial cells, such as diminished leukocyte adhesion and enhanced antioxidative defence were reported before^40–46^. In contrast to the sometimes fluctuating gene expression responses, changes in protein levels of Cx40 were coherent, demonstrating dose-dependent downregulation, significant from low dose (0.1 Gy) at different time points in TICAE and TIME cells. For Cx43, gene expression and protein levels grossly corresponded and demonstrated a dose-dependent elevation at different time points in TICAE and TIME cells. We furthermore found radiation-induced elevation of phosphorylated and hyperphosphorylated Cx43 forms for both cell types. Interestingly, a previous study showed that endothelial Cxs have a distinct temporal expression pattern over time in non-irradiated conditions^47^. In summary, as indicated in PCA (Fig. 6), there is a dose-dependent response in Cx40 and Cx43 after radiation exposure, giving the strongest response at 5 Gy. Additionally, the profile of the irradiated cell responses neutralized after 14 d for 0.1 Gy, while 0.5 Gy and higher gave a more permanent change within the 14 d observation window.

As radiotherapy is delivered to the tumour in multiple radiation fractions, assessing the effect of fractionated irradiation on vascular endothelial cells is of clinical importance. As indicated in PCA (Supplementary Fig. S3) both single and fractionated irradiation induced the dose-dependent response in Cx40 and Cx43, with persistent changes for 5 Gy condition. However, there are some differences between single and fractionated irradiation response that are more pronounced in TIME cells, as indicated in Supplementary Table S2, suggesting that fractionated irradiation response is cell type dependent. However, it worth mentioning that the comparison between single and fractionated irradiation is limited to the radiation response after normalizing the controls (fold changes). The overall observed decreased expression of atheroprotective Cx37 (gene) and Cx40 (gene/protein), and the increase in the proatherogenic Cx43 (gene/protein) induced by radiation exposure may positively modulate susceptibility to atherosclerosis as delineated in the introduction.

In addition to alterations at the gene and protein level, we also observed functional changes at the level of both gap junctions and hemichannels. Overall, it was clear that irradiation increased gap junctional communication as assessed by dye coupling, which was most obvious at 72 h post single and fractionated irradiation in both TICAE and TIME cells. Taken together, the prominent presence of Cx43 in vascular endothelial cells of the major arteries^23^, combined with the variable observations for Cx37 gene alterations and the decreased level of Cx40 protein, the observed increase in gap junctional coupling is likely caused by the increased Cx43 expression, possibly in combination with the increase in phosphorylated forms that act to enhance gap junctional communication. At the level of hemichannels, we used ATP release and propidium iodide dye uptake assays to estimate functional alterations^48–50^. To further increase the robustness of the responses, we used TAT-Gap19, a Cx43 hemichannel inhibitor not inhibiting gap junction channels^51^, to determine whether the responses were Cx43 hemichannel-related. Supplementary Table S1 clearly demonstrates an overall increased hemichannel function (ATP release and dye uptake) in the two cell lines for 5 Gy at the 72 h time point. Irradiation with 5 Gy also showed early 1 h responses in the two cell types. In addition, fractionated irradiation increased hemichannel function (ATP release and dye uptake) mainly at 5 Gy. Thus, hemichannel function was, as gap junctional function, increased in response to single and fractionated irradiation. As concluded for gap junctions, the increased hemichannel function may relate to increased post irradiation Cx43 expression and its phosphorylated forms. However, unlike gap junctions that are normally open, hemichannels are normally closed and need a trigger to open the channels. Connexin hemichannels open in response to physiological and pathological triggers, including changes in membrane potential, changes in intracellular or extracellular Ca^2+^ concentration, redox alterations, post-translational alterations including phosphorylation and nitrosylation, ischemia and inflammation (reviewed in^23^). Cx43 hemichannel opening, as reported by ATP release assays, has also been demonstrated in B16 melanoma cells in response to γ-rays^49^. X-rays interact with water to form ROS, which acts as a trigger for Cx43 hemichannel opening^52^. In addition, X-rays are known to interact with cysteine, oxidizing it to cystine^53^. Cx43 has 3 cysteines in each extracellular loop and several Cys residues inside the cell, including the C-terminal tail that is crucial for regulating hemichannel function (reviewed in^23^). S-nitrosylation, possibly by NO acting at CT-located Cys residues is again a known trigger of hemichannel opening^54^. Uncontrolled hemichannel opening results in excessive entry of Na^+^ and Ca^2+^, ATP leakage and loss of cell-essential metabolites, which in turn can trigger intracellular events, such as inflammation, NO production and activation of apoptotic caspases^49,55,56^. Several studies demonstrated that excessive hemichannel opening contributes to different pathological processes including spread of bystander signalling such as inflammation and oxidative stress, blood-brain barrier opening and cardiac ischemia/reperfusion injury^57–60^. Furthermore, ATP released via hemichannels and other pathways is a danger signal to the immune system that triggers intracellular Ca^2+^ signalling and is known to activate the NLRP3 inflammasome^61–63^. DNA damage, oxidative stress, apoptosis and inflammation are known mechanism to be involved in the pathogenesis of radiation-induced atherosclerosis. These radiation damaging responses may spread through gap junction intercellular communication and paracrine signalling mediated via hemichannel into neighbouring cells, possibly amplifying endothelial cell damage^35,57,64–68^.

## Conclusion

In conclusion, to the best of our knowledge, this is the first study to show that exposure of coronary artery and microvascular endothelial cells to single or fractionated X-rays, induced an acute and persistent dose-dependent decrease of atheroprotective Cx40 and increase of the proatherogenic Cx43 gene and protein levels. In addition, such radiation exposures increased gap junctional communication and induced acute and long-lived hemichannel opening, the latter considered as a pathological condition. We hypothesize that radiation-induced increased endothelial gap junctional coupling and hemichannel function may lead to endothelial dysfunction, which is an early marker for atherosclerosis. Unraveling the role of connexin-based communication pathways in radiation-induced atherosclerosis may further improve our understanding of these multifunctional proteins as a potential target to prevent radiation-induced complications.

## Methods

### Cell culture

We used two human endothelial cell lines: hTERT telomerase immortalized human coronary artery endothelial cells (TICAE) from the European Collection of Authenticated Cell Cultures (ECACC; HCAECs Cat. No: 300-05), and telomerase immortalized human dermal microvascular endothelial cells (TIME) from the American Type Cell Culture (ATTC). TICAE cells are not tumorigenic and they display all major endothelial phenotypic markers, such as, PECAM1, vonWillebrand factor and cadherin-5 (unpublished data). In addition, they have a response to radiation exposure similar to their primary counterparts^69^. TIME cells are positive for CD31, capable of taking up Low Density Lipoprotein (LDL), and are karyotypically, morphologically, and phenotypically similar to the primary parent cells (data provided by ATTC).

TICAE and TIME cells were grown in MesoEndo Cell Growth Medium (Sigma-Aldrich Co. LCC, Diegem, Belgium). The passage number that was used in all the experiments (for controls and irradiated conditions) is between passages 26 until passage 32. The cells were kept in a humidified incubator at 37 °C supplemented with 5% CO_2_ and split every two to three days with a 0.05% trypsin solution supplemented with 0.02% ethylenediaminetetraacetic acid (EDTA) (Life Technologies, Merelbeke, Belgium). Cells were counted via Moxi Z Mini Automated Cell Counter (ORFLO Technologies, Ketchum, ID, USA). Cells were not sub-cultured during the course of the experiments, but medium was changed twice/thrice per week for 7 days and 14 days single irradiation experiments, and for 7 days fractionated irradiation experiment.

### Irradiation

Both TICAE and TIME cells were irradiated at 100% confluence with a vertical point source X-ray beam using a Xstrahl RX generator (Camberley, UK; 320 kV, 12 mA, 3.8 mm Al and 1 mm Cu). X-rays doses (0.1, 0.5 and 5 Gy) were delivered to the cells either in one session (‘single irradiation’) or in a fractionated manner with three X-rays doses administered over three consecutive days (0.033 and 1.67 Gy/fraction/day), leading to an accumulative dose of 0.1 and 5 Gy (‘fractionated irradiation’). The dose rate used was 0.5 Gy/min for both single and fractionated exposure. Dosimetry was applied for all the experiments to ensure uniformity of dose and dose rate delivered, following ISO 4037 and ISO-17025 recommendations. Non-irradiated controls for all experiments were treated with the same conditions like irradiated samples, except they are sham-irradiated (0 Gy control).

### Gene expression analysis via RT-qPCR

TICAE and TIME cells were seeded in 6-well plates at a density of 2.5 × 10^5^ cells/well in five biological replicates. After three/four days, cells reached 100% confluence. The medium was changed before irradiation with 2 ml medium/well for single irradiation experiment, and 3 ml medium/well for fractionated irradiation experiment. Without refreshing the medium, cells were harvested 6, 24, 48 and 72 h post irradiation (p.i.). For the cells harvested 7 and 14 days p.i., the medium was refreshed every two/three days. After harvest, cells were stored in RLT Plus buffer and the total RNA was isolated with the RNeasy Mini Kit (QIAGEN, Venlo, The Netherlands) according to the manufacturer’s instructions. Concentration and quality of RNA were assessed spectrophotometrically using NanoDrop 2000c (Applied Biosystems, Thermofisher Scientific, Waltham, Ma, USA).

The GoScript Reverse Transcription System (Promega, Leiden, The Netherlands) was used to prepare the complementary DNA (cDNA) by adding 1 μL Random Nucleotide and 1 μL Oligo (dT) primers in 21 μL reactions. After denaturation, a mixture of 8 μL GoScript 5x Reaction Buffer, 6 μL magnesium chloride (MgCl_2_), 2 μL PCR Nucleotide Mix, 1 μL Recombinant RNasin Ribonuclease Inhibitor and 2 μL GoScript Reverse Transcriptase was added to each sample.

Two technical replicates for each biological replicate were prepared and quantitative polymerase chain reaction (qPCR) was performed in Fast Optical 96-Well Reaction Plates (Applied Biosystems, Gaasbeek, Belgium) using MESA GREEN qPCR MasterMix Plus for SYBR Assay Low ROX (Eurogentec, Seraing, Belgium) on a 7500 Fast Real-Time PCR System (Applied Biosystems, Thermofisher Scientific, Waltham, Ma, USA). Amplification occurred at the following cycling conditions: 5 minutes 95 °C, 40 cycles of 3 seconds at 95 °C, and 45 seconds at 60 °C, followed by the generation of a dissociation curve to verify amplification specificity. Reactions contained 12.5 μL 1x Fast SYBR Green Master Mix, 25 mM forward primer and 25 mM reverse primer (sequences selected from literature or qPrimerDepot database (https://primerdepot.nci.nih.gov/)), 20 ng of cDNA template (for all the conditions of all the experiments) and nuclease-free water in a total amount of 25 μL^70^. To evaluate primer efficiency, a standard curve was generated using a two-fold dilution series of a sample over at least five dilution points. In addition, the specificity of the primers was confirmed by performing gel electrophoresis. Primer sequences and efficiencies are shown in Table 1. All measurements were performed in duplicate and the mean of two values from each sample was used in further analyses. The mathematical method of Pfaffl was used to quantify the gene expression^71^. Housekeeping genes, *INPP1* and *PGK1* were selected to obtain sample-specific normalization factors.

**Table 1:** Forward and reverse primers used to determine gene expression levels via RT-qPCR. RT-qPCR: reverse transcription quantitative polymerase chain reaction, INPP: inositolpolyphosphate-1-phosphatase, PGK1: phosphoglycerate kinase 1, Cx: connexin, FW: forward, RV: reverse.

	Gene	Sequence (5′-3′)	Primer efficiency
Housekeeping genes	*INPP1*	FW: CTCCTGCTCTGTCCTCATCC RV: CTCCCGGAGGATATCTGACA	96%
	*PGK1*	FW: CAAGAAGTATGCTGAGGCTGTCA RV: CAAATACCCCCACAGGACCAT	102%
Genes of interest	*Cx37 (GJA4)*	FW: GGTGGGTAAGATCTGGCTGA RV: GGCCGTGTTACACTCGAAAT	91.7%
	*Cx40 (GJA5)*	FW: CAGGGAACAGATGCCAAAAC RV: AGTTGGAGAAGAAGCAGCCCA	108.6%
	*Cx43 (GJA1)*	FW: TCTGAGTGCCTGAACTTGC RV: ACTGACAGCCACACCTTCC	96.1%

### Protein extraction and western blot analysis

TICAE and TIME cells were seeded in 6-well plates at a density of 2.5 × 10^5^ cells/well in four to six biological replicates. After three/four days, cells reached 100% confluence. The medium was changed before irradiation with 2 ml medium/well for single irradiation experiment, and 3 ml medium/well for fractionated irradiation experiment. To extract proteins, 200 μL of RIPA lysis buffer (Roche, Brussels, Belgium), consisting out of 150 mM NaCl, 50 mM Tris-HCl pH 7.4, 1% NP-40/IGEPAL CA-630, 0.5% sodium deoxycholate, 0.1% SDS, a phosphatase tablet and protease inhibitor tablet (Roche, Brussels, Belgium), was added to 10^6^ cells. Next, cells were homogenized for 30 seconds with a tissue lyser II device (Qiagen, Antwerp, Belgium). The protein concentration was determined with the bicinchoninic acid (BCA) Protein Assay kit (Sigma-Aldrich Co. LLC, Diegem, Belgium). Subsequently, for all the conditions of all the experiments, 10 μg of proteins (except for Cx40 TIME, 20 μg was used) were supplemented with Laemmli buffer (1/4 of the total volume) (Bio-Rad, Temse, Belgium), β-mercaptoethanol (1/10 of the Laemmli buffer) (Sigma-Aldrich Co. LLC, Diegem, Belgium) and heated at 95 °C for 5 minutes. Electrophoresis was performed at 160 volt and the separated proteins were transferred to a nitrocellulose membrane (Applied Biosystems, Thermofisher Scientific, Waltham, Ma, USA) using the iBlot dry transfer system (Invitrogen™ Thermo Fisher Scientific, Ninove, Belgium). The membranes were blocked for 2–3 h at room temperature using either 5% non-fat dry milk (NFDM) (Bio-Rad, Temse, Belgium) or 5% BSA (Sigma-Aldrich Co. LLC, Diegem, Belgium) (Table 2). Afterwards, membranes were incubated overnight at 4 °C with the appropriate primary antibody (Table 2). After washing, the membrane was incubated for 45 minutes at room temperature with the appropriate horse radish peroxidase (HRP)-conjugated secondary antibodies (Life Technologies, Merelbeke, Belgium) (Table 2). The HRP-immunoreactive bands were visualized with the ECL detection kit (Bio-Rad, Temse, Belgium) and scanned using the Fusion Fx imaging device (Vilber Lourmat, Eberhardzell, Germany). Signals were quantified densitometrically using Bio1D analysis software (Vilber Lourmat, Eberhardzell, Germany) and expressed as relative values (i.e. normalized to the corresponding vinculin signal of the same membrane). Cx37 protein level couldn’t be detected with 10–30 µg protein concentration due to the low endogenous level.

**Table 2:** List of primary antibodies, secondary antibodies and blocking buffer used for western blot analysis are listed.

Target	Blocking buffer	Antibody	Concentration	Species	Reference
Cx37	5% NFDM	Primary	1/500	Rabbit	Life Technologies (Belgium)
		Secondary	1/10 000	Goat	Life Technologies (Belgium)
Cx40	5% BSA	Primary	1/500	Goat	Santa Cruz (Germany)
		Secondary	1/10 000	Rabbit	Life Technologies (Belgium)
Cx43	5% NFDM	Primary	1/1 000	Rabbit	Sigma-Aldrich Co.(Belgium)
		Secondary	1/20 000	Goat	Life Technologies (Belgium)
Vinculin	5% NFDM	Primary	1/1 000	Mouse	Santa Cruz (Germany)
		Secondary	1/20 000	Goat	Life Technologies (Belgium)

Cx: connexin, NFDM: non-fat dry milk, BSA: bovine serum albumin.

### Scrape loading and dye transfer

The scrape loading and dye transfer assay (SLDT) was used to assess gap junctional function after IR exposure. TICAE and TIME cells were seeded in a 24-well plate at a density of 1 × 10^5^ cells/well in four to six biological replicates. Three days later, cells reached 100% confluence and the medium was refreshed before irradiation with 1 ml/well for single irradiation experiment and 1.5 ml for fractionated irradiation experiment. At different time points after irradiation (6 and 72 h post single irradiation, or 72 h post fractionated irradiation) the cells were washed 3 times with a scrape loading and dye transfer (SLDT) buffer (137 mM NaCl, 5.36 mM KCl, 0.81 mM MgCl_2_, 5.55 mM MgCl_2_.6H_2_O, 25 mM HEPES, pH 7.4). Then, the cells were incubated with a SLDT solution composed of SLDT buffer and 400 μM 6-carboxyfluorescein (6-CF, 0.376 kDa) (Sigma-Aldrich Co. LCC, Diegem, Belgium) for one minute. A vertical scratch was made in the middle of each well, using a 20G needle (Becton Dickinson, Erembodegem, Belgium). After another one minute incubation, the cells were washed 6 times with HBSS supplemented with 25 mM HEPES (Sigma-Aldrich Co. LCC, Diegem, Belgium). Carbenoxolone (Sigma-Aldrich Co. LCC, Diegem, Belgium; 50 µM) was used as a control for blocking gap junctional coupling. After an incubation time of 10 minutes, the cells were visualized with an Eclipse Ti automated inverted wide-field epifluorescence microscope (Nikon, Brussels, Belgium) equipped with a 5 × dry objective (Plan Fluor, numerical aperture 0.6) and a Nikon TE2000-E camera controlled by the NIS Elements software. The relative area of 6-CF transfer was calculated using FIJI software. By analyzing the area of the dye diffusion from the first line of cells to adjacent cells, the gap junctional coupling was determined.

### Extracellular ATP measurements

Hemichannels are an ATP release pathway and we therefore determined extracellular ATP after irradiation. To that purpose, TICAE and TIME cells were seeded in a 96-well plate at a density of 1 × 10^5^ cells/well and 0.3 × 10^5^ cells/well, respectively, in six to eight biological replicates. Three days later, cells reached 100% confluence. At 30 minutes before irradiation, cells were refreshed with 100 μl medium alone or with medium supplemented with 100 μM TAT-Gap19 (Genosphere Biotechnologies, Paris, France) to block the Cx43 hemichannels. For fractionated irradiation, cells were refreshed with 150 μl medium/well. At different time points after irradiation (1, 6 and 72 h), 1:5 ATP assay mix dissolved in ATP mix dilution buffer (Sigma-Aldrich Co. LCC, Diegem, Belgium) was added to each well. Extracellular ATP release was assessed by measuring the luminescence signal received after oxidation of luciferin and ATP to oxyluciferin and AMP using the CLARIOstar microplate reader (BMG Labtech, Temse, Belgium). Experiments were corrected for baseline ATP signal in medium in the absence or presence of TAT-Gap19.

### Dye uptake assay

Hemichannel opening was also investigated by dye uptake studies making use of the hemichannel permeable fluorescent tracer propidium iodide (PI; MW 668.4 Da). In addition, dextran fluorescein 10 kDa dye was used to assess membrane integrity and occurrence of cell death. PI stains cells with open hemichannels, late apoptotic and necrotic cells, while dextran fluorescein only stains late apoptotic and necrotic cells. Therefore, PI-positive and dextran fluorescein-negative cells are a measure for hemichannel opening while dextran fluorescein-positive cells are a measure for dead cells.

TICAE and TIME cells were seeded in a 24-well plate at a density of 1.25 × 10^5^ and 1.5 × 10^5^ cells/well, respectively, in six biological replicates. Three days later, cells reached 100% confluence. Thirty minutes before irradiation, cells were refreshed with 1 ml medium alone or with medium supplemented with 100 μM TAT-Gap19 (Genosphere Biotechnologies, Paris, France). For fractionated irradiation, cells were refreshed with 1.5 ml medium/well. At different time points after irradiation (1, 6 and 72 h), cells were washed twice with HBSS supplemented with 25 mM HEPES (Sigma-Aldrich Co. LCC, Diegem, Belgium) (HBSS-HEPES). Afterwards, the cells were incubated with 1 mM PI and 200 μM dextran fluorescein (10 kDa) (Life Technologies, Merelbeke, Belgium), dissolved in HBSS-HEPES for 5 minutes followed by 5 times washing with HBSS-HEPES. Finally, the fluorescence was measured using an IncuCyte ZOOM system (Essen BioScience, Ann Arbor, Michigan, USA) by using different channels (TRITC, FITC and phase contrast) and a 10x objective Fluorescence signal was normalized to cell number.

### Principal component analysis (PCA)

In order to have an overview of the results, principal component analysis (PCA) was performed for Cx40 and Cx43 gene expression and protein level (assessed by RT-qPCR and western blot) for both TICAE and TIME cells after single irradiation over the 6 h to 14 d p.i. time period. As the same sample was not used for both qPCR and western blot analysis, and the number of replicates were varying from 4–6 replicates, the means of the various replicates for each time point/radiation dose were calculated and PCA was performed using the prcomp command in R software (v 3.4.3). Both first and second components were plotted in the horizontal and vertical axes, respectively. For each time point and each radiation dose, the gene expression/protein level profile was averaged and analyzed using two-dimension PCA.

### Statistical analysis

All experiments were analyzed with a nonparametric Mann-Whitney T-test. The results were considered statistically significant when p < 0.05. Data are presented as mean ± standard error of the mean. Statistical analysis was done with GraphPad Prism 5.01 (GraphPad Software Inc., La Jolla, CA 92037 USA). Occasional exclusion of outlier data points were done using Grubbs’ test.

## Supplementary information

Supplementary tables and figures

## Data Availability

The datasets generated during and/or analysed during the current study are available from the corresponding author on reasonable request.
