# Supplementary material for: Single and fractionated ionizing radiation induce alterations in endothelial connexin expression and channel function
Source: Sci Rep. 2019 Jun 20;9:4643. doi: 10.1038/s41598-019-39317-9 (PMC6584668; doi:10.1038/s41598-019-39317-9)
Supplement: Supplementary file 1 — Supplementary tables and figures [file 41598_2019_39317_MOESM1_ESM.pdf]

Single and fractionated ionizing radiation induce alterations in endothelial connexin expression and channel function

Raghda Ramadan[1,2], Els Vromans[3], Dornatien Chuo Anang[4], Elke Decrock[2], Mohamed Mysara[5], Pieter Monsieurs[5], Sarah Baatout[1,6], Luc Leybaert[2≠], An Aerts[1≠] *

[1]*Radiobiology Unit, Belgian Nuclear Research Centre (SCK•CEN), Mol, Belgium;*
[2]*Department of Basic Medical Sciences, Physiology group, Ghent University, Ghent, Belgium;*
[3] *Centre for Environmental Health Sciences, Hasselt University, Hasselt, Belgium;*
[4]*Biomedical Research Institute and transnational university of Limburg, Hasselt University, Hasselt, Belgium;*
[5]*Microbiology Unit, Belgian Nuclear Research Centre (SCK•CEN), Mol, Belgium;*
[6]*Department of Molecular Biotechnology, Ghent University, Ghent, Belgium,*

≠ Share senior authorship.
E-mail: an.aerts@sckcen.be; luc.leybaert@ugent.be

**E-mail: an.aerts@sckcen.be*

Supplementary Table S1. Summary of single irradiation effects on gap junction and hemichannel function.

		1 h	6 h	72 h
Gap junctional dye coupling	TICAE	-	5 Gy ↑	0.1 & 5 Gy ↑
	TIME	-	-	0.1 & 5 Gy ↑
*Hemichannel ATP release	TICAE	-	5 Gy ↑	5 Gy ↑
	TIME	5 Gy ↑	0.1 & 5 Gy ↑	0.1 & 5 Gy ↑
*Hemichannel dye uptake	TICAE	5 Gy ↑	-	5 Gy ↑
	TIME	-	-	0.1 & 5 Gy ↑

*Only TAT-Gap19 inhibitable responses shown, -: indicates no significant effect, ↑: indicates an increase.

Supplementary Table S2. Distinct effects of fractionated irradiation versus single irradiation found in this study.

		24 h	72 h	7 d
Gene expression	TICAE	-	-	↑Cx40 at 5 Gy
	TIME	↓Cx40 at 5 Gy<br>↓ Cx43 at 0.1 & 5 Gy	-	↑Cx37 at 0.1 & 5 Gy<br>↓Cx40 at 0.1 Gy<br>↑Cx43 at 0.1 & 5 Gy
Protein level	TICAE	-	-	↑Cx40 at5 Gy
	TIME	-	-	↑Cx40 at5 Gy
Gap junctional dye coupling	TICAE	-	-	-
	TIME	-	↓0.1 Gy	-
Hemichannel ATP release	TICAE	-	↑ 0.1 Gy	-
	TIME	-	-	-
Hemichannel dye uptake	TICAE	-	↑ 5 Gy	-
	TIME	-	-	-

-: indicates no significant effect, ↑: indicates an increase, ↓: indicates a decrease.

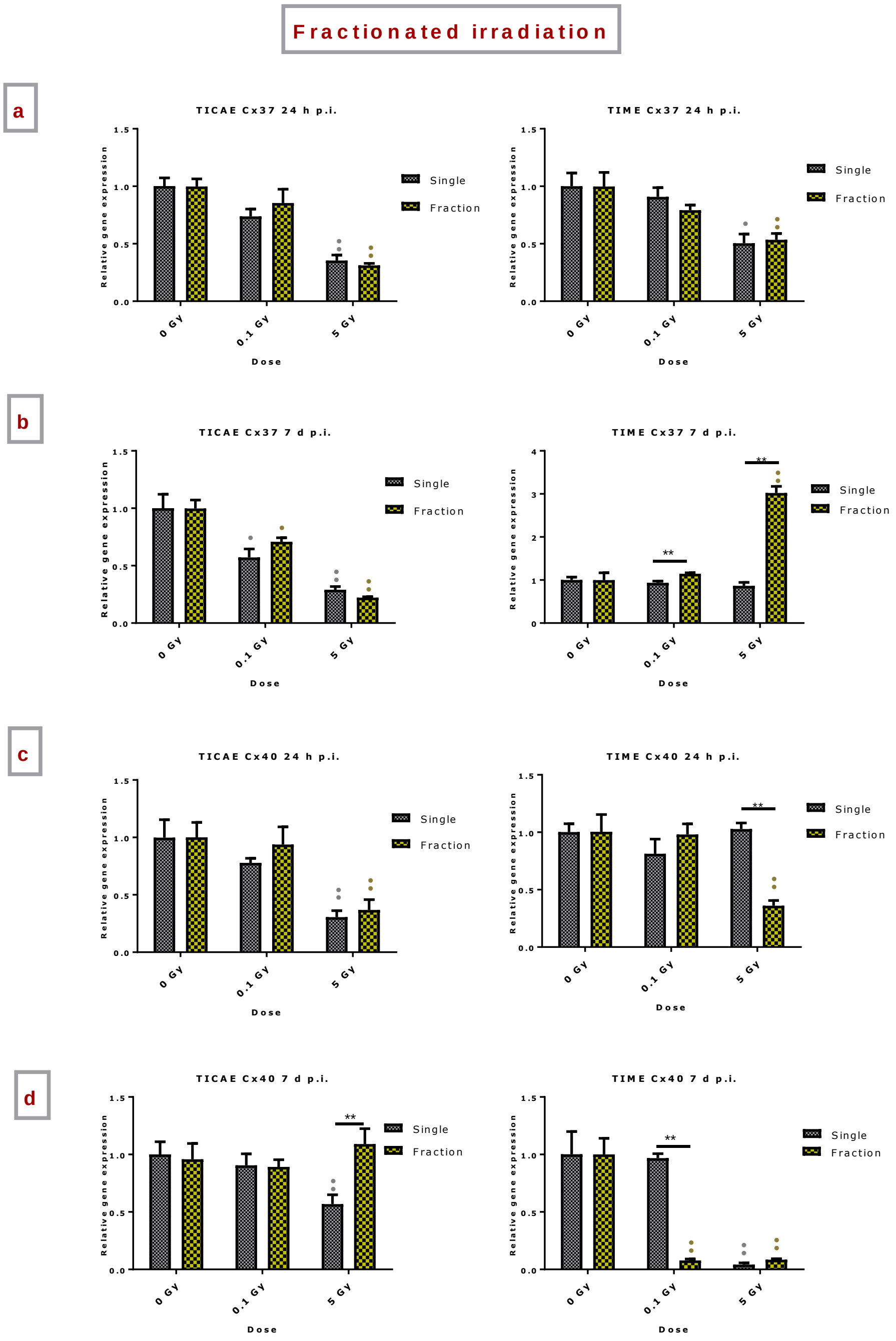

Supplementary Figure S1. The effect of single and fractionated irradiation on Cx37 and Cx40 gene expression at 24 h and 7 d post irradiation exposure. Cx37 and Cx40 relative gene expression (fold change) were assessed at (a and c) 24 h and (b and d) 7 d after single and fractionated X-ray exposure (0.1 and 5 Gy) in TICAE (left side) and TIME cells (right side). Comparison between single and fractionated irradiation is limited to the radiation response after normalizing the controls. Data were analyzed with a nonparametric Mann-Whitney T-test. Values represent average ± SEM of five biological replicates. * indicates the statistical differences between single and fractionated irradiation for the same radiation dose. • indicates the statistical differences for either single or fractionated irradiation compared to their respective 0 Gy controls. */•: $p<0.05$; **/••: $p< 0.01$.

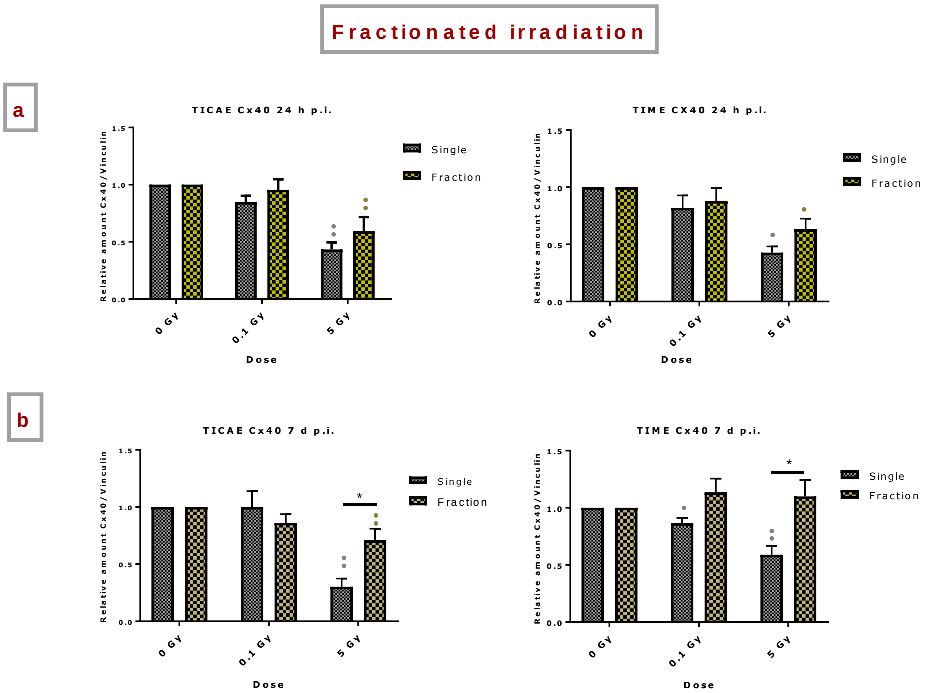

Supplementary Figure S2. The effect of single and fractionated irradiation on Cx40 protein levels in TICAE and TIME cells (A) 24 h and (B) 7 d post irradiation. Cx40 protein levels were assessed (A) 24 h and (B) 7 d after a single and fractionated X-ray exposure (0.1 and 5 Gy) in TICAE and TIME cells. These data were analyzed with a nonparametric Mann-Whitney T-test. The values represent the average ± SEM of five to six biological replicates. * indicates the statistical differences between single and fractionated irradiation for the same radiation dose. • indicates the statistical differences for either single or fractionated irradiation compared to their respective 0 Gy controls. */•: P<0.05; **/••: P< 0.01.

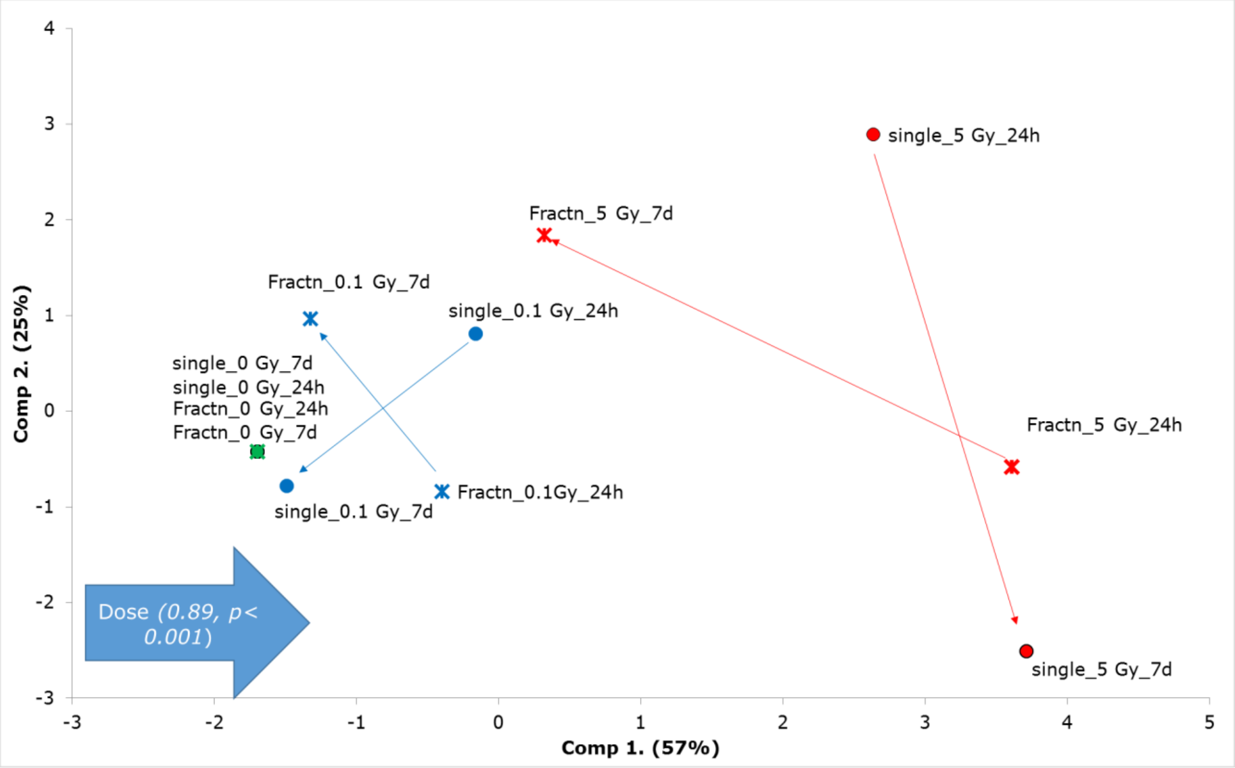

Supplementary Figure S3. Two dimensional principal component analysis (PCA) incorporating Cx40 and Cx43 gene expression and protein level for single and fractionated irradiation at 24 h and 7 d p.i. in TICAE and TIME cells. Such analysis indicated a dose-dependent separation between the radiation doses used (0.1 and 5 Gy) in both single and fractionated irradiation, which significantly shifted the PCA profiles along the positive side of the first component axis (*p<0.001*), reflecting a dose-dependent response in Cx40 and Cx43 gene expression and protein level after both single and fractionated radiation exposure. The colors correspond to the radiation doses, where 0.1 and 5 Gy were colored blue and red, respectively.

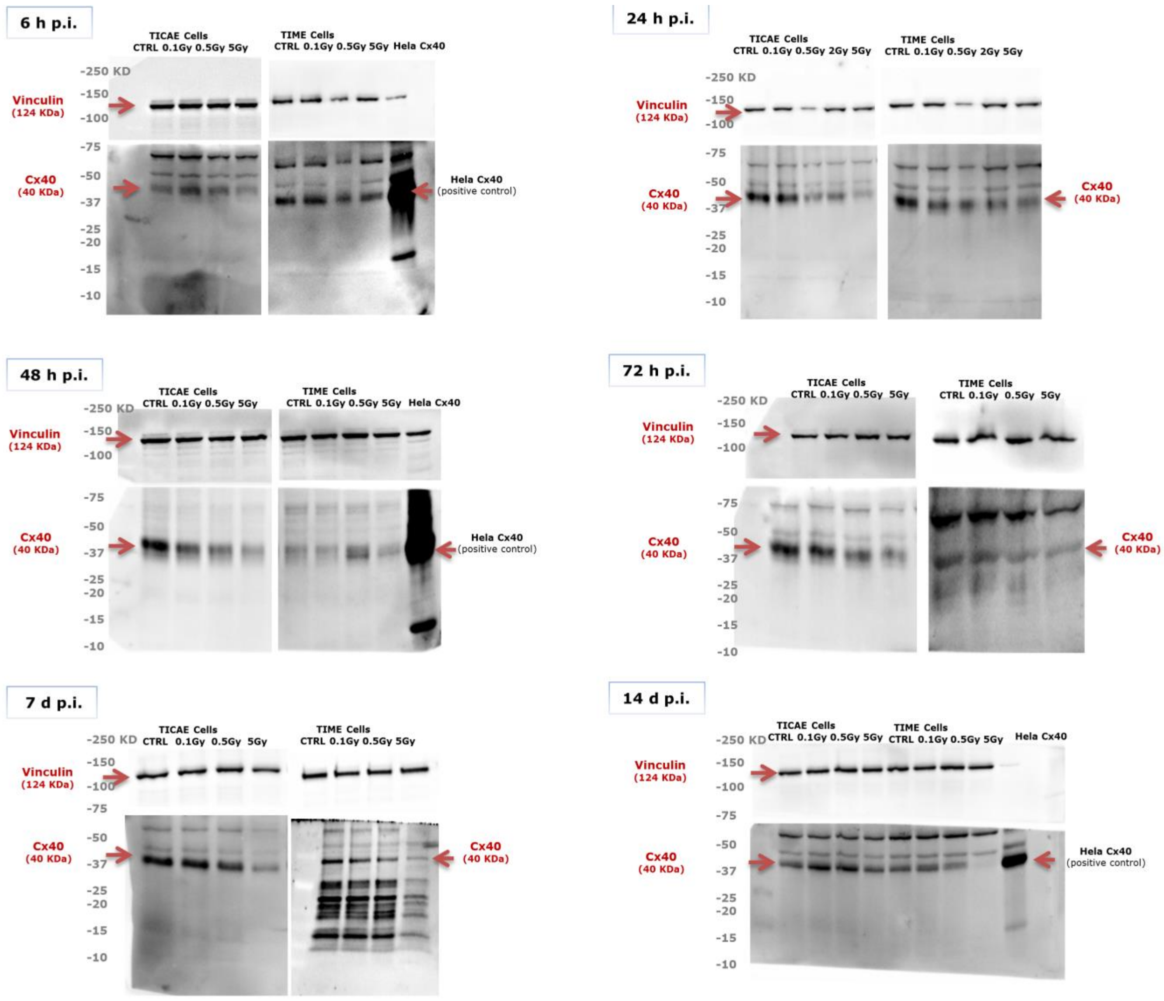

6 h p.i.
TICAE Cells
CTRL 0.1Gy 0.5Gy 5Gy
TIME Cells
CTRL 0.1Gy 0.5Gy 5Gy Hela Cx40
-250 KD
-150
-100
-75
-50
-37
-25
-20
-15
-10
Vinculin
(124 KDa)
Cx40
(40 KDa)
Hela Cx40
(positive control)
24 h p.i.
TICAE Cells
CTRL 0.1Gy 0.5Gy 2Gy 5Gy
TIME Cells
CTRL 0.1Gy 0.5Gy 2Gy 5Gy
48 h p.i.
72 h p.i.
7 d p.i.
14 d p.i.

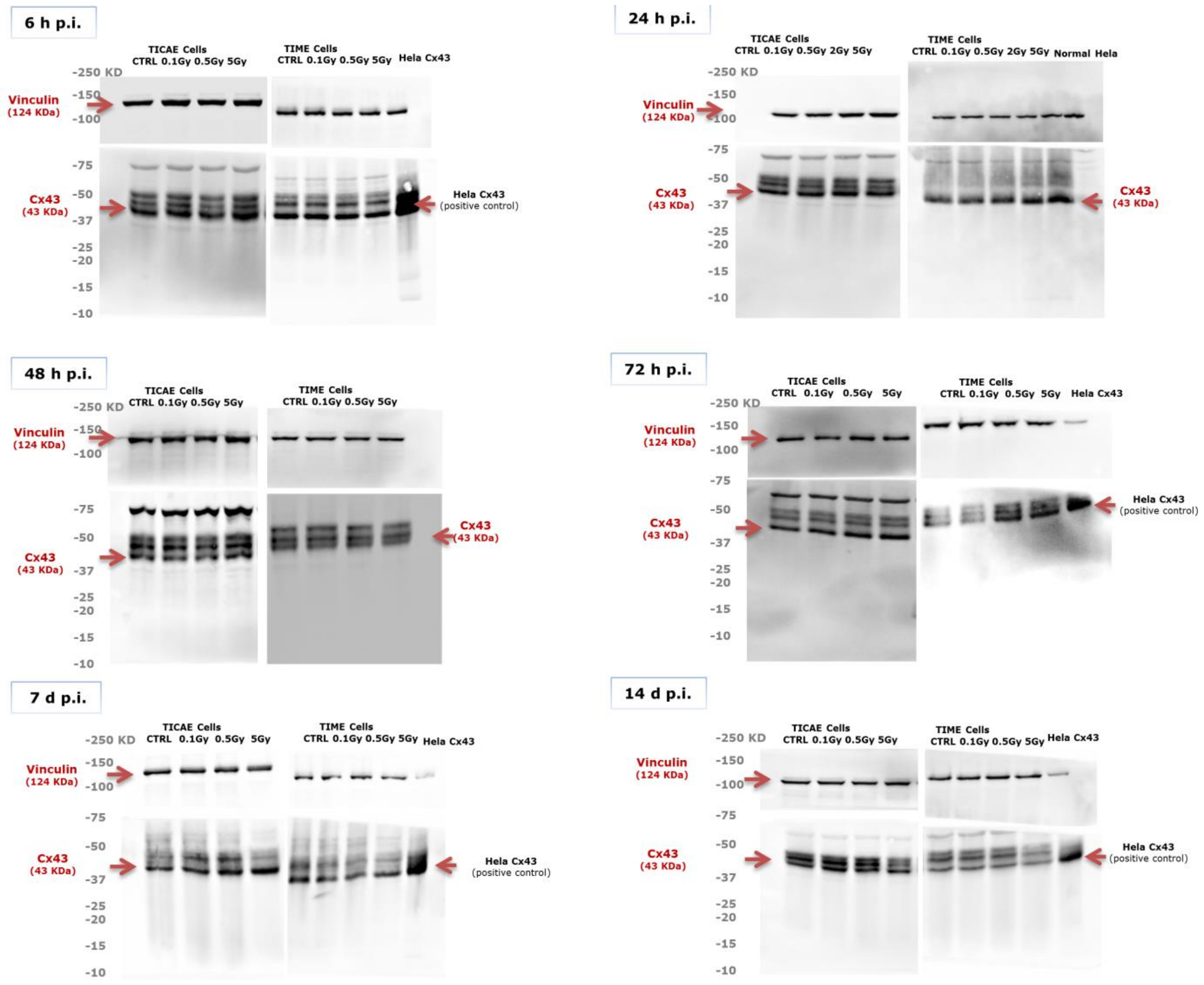

Supplementary Figure S4: Full length blots of Cx40 and Cx43 in TICAE and TIME cells shown in Figure 2. For all the blots, Vinculin was ran on the same gel and the membranes were cut between 75 and 100 KD to image Vinculin and Cx40/CX43 separately. Signals were normalized to the corresponding vinculin signal of the same membrane and quantified densitometrically using Bio1D analysis software. Some gel images were cropped to remove samples that were ran on the same gel, but were not used in Figure 2 of this study.

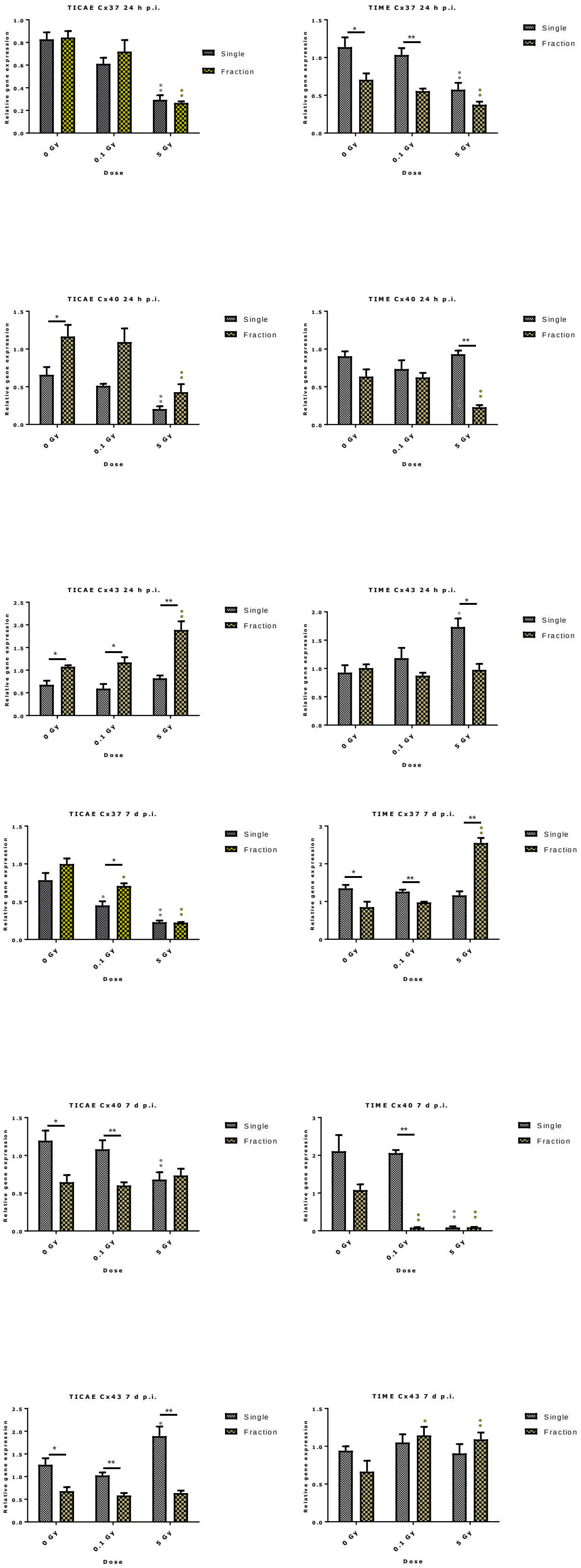
TICAE Cx37 24 h p.i.
TIME Cx37 24 h p.i.
TICAE Cx40 24 h p.i.
TIME Cx40 24 h p.i.
TICAE Cx43 24 h p.i.
TIME Cx43 24 h p.i.
TICAE Cx37 7 d p.i.
TIME Cx37 7 d p.i.
TICAE Cx40 7 d p.i.
TIME Cx40 7 d p.i.
TICAE Cx43 7 d p.i.
TIME Cx43 7 d p.i.
Relative gene expression
Dose
0 Gy
0.1 Gy
5 Gy
Single
Fraction

Supplementary figure S5: The effect of single and fractionated irradiation on Cx37, Cx40 and Cx43 gene expression at 24 h and 7 d post irradiation, in TICAE (left panel) and TIME (right panel) without performing normalization of the controls. Normalization of the control of different time points and for radiation regimen (single and fractionated) were performed in figure 1 and Supplementary Figure S1 to avoid technical and experimental variations, as the experiments of different time points were not treated as the same time (even though the measurements were presumably carried out under identical circumstances), and they were ran on different qPCR runs. These data were analyzed with a nonparametric Mann-Whitney T-test. The values represent the average ± SEM of five to six biological replicates. * indicates the statistical differences between single and fractionated irradiation for the same radiation dose. • indicates the statistical differences for either single or fractionated irradiation compared to their respective 0 Gy controls. */•: $P<0.05$; **/••: $P< 0.01$.
